# Prevalence and Outcomes of Depression, Obstructive Sleep Apnea, and Concurrent Anxiety (DOCA) in Stroke Survivors: Insights From a Nationwide Study

**DOI:** 10.7759/cureus.41968

**Published:** 2023-07-16

**Authors:** Urvish K Patel, Alankruta Rao, Gurpal Singh D Manihani, Neel Patel, Cilgy George, Jai Sriram Vijayakumar, Sekharamahanti H Evangeline, Mohammad R Alam, Kulbir Ghuman, Stephan Z Francis, Ishani Pandya, Chandrakanth Reddy, Tapan Parikh, Shamik Shah

**Affiliations:** 1 Department of Public Health and Neurology, Icahn School of Medicine at Mount Sinai, New York, USA; 2 Department of Medicine, Krishna Institute of Medical Sciences, Karad, IND; 3 Department of Neurology, Rajagiri Hospital, Aluva, IND; 4 Department of Medicine, Dhanalakshmi Srinivasan Medical College and Hospital, Perambalur, IND; 5 Department of Medicine, Ganni Subba Lakshmi (GSL) Medical College, Rajahmundry, IND; 6 Department of Internal Medicine, Arghakhanchi District Hospital, Sandhikharka, NPL; 7 Department of Medicine, Dayanand Medical College and Hospital, Ludhiana, IND; 8 Department of Internal Medicine, Saba University School of Medicine, The Bottom, BES; 9 Department of Medicine, Grodno State Medical University, Grodno, BLR; 10 Department of Radiology, M.N. Raju Medical College and Hospital, Sangareddy, IND; 11 Department of Psychiatry, Feinberg School of Medicine, Chicago, USA; 12 Department of Neurology, Stormont Vail Health, Topeka, USA

**Keywords:** cerebro-vascular accident (stroke), obstructive sleep apnea (osa), mood and anxiety, post stroke recovery, doca, concurrent anxiety, depression

## Abstract

Background: Many individuals will also experience psychological side effects after a stroke episode, such as symptoms of depression, anxiety (generalized anxiety disorder (GAD)), and/or specific phobias, considerably decreasing their quality of life (QOL). Objective: This study aimed to evaluate the prevalence of depression, obstructive sleep apnea (OSA), and concurrent anxiety (DOCA) and their outcomes (morbidity, disability (All Patient Refined Diagnosis Related Group (APRDRG) - loss of function), and discharge disposition) among acute ischemic stroke (AIS) hospitalizations. Methods: A cross-sectional study used the National Inpatient Sample (NIS) from 2003-2017. Adults with hospitalizations with AIS were extracted, and DOCA was identified using ICD-9/10-CM codes. Weighted analysis using a chi-square test and mixed-effect multivariable survey logistic regression was used to assess the prevalence and role of DOCA in predicting outcomes. Results: Out of 5,690,773 AIS hospitalizations, 2.7%, 3.1%, and 4.4% had depression, OSA, and GAD, respectively. In AIS patients, females had a higher prevalence of depression (3.4% vs. 2.3%) and GAD (5.9% vs. 3.0%) and a quality of life lower prevalence of OSA (2.2% vs 4.4%) in comparison to males (p<0.0001). Caucasians had a higher prevalence of depression, OSA, and GAD in comparison to others (African Americans/Hispanics/Asians/Native Americans). Depressed patients had a higher prevalence of morbidity (9% vs. 8% vs 5% vs. 7%), disability (46% vs. 46% vs. 35% vs. 37%), transfer to non-home (69% vs. 58% vs. 61% vs. 63%) in comparison with OSA, GAD, and non-DOCA patients, respectively (p<0.0001). Depression was associated with a 40% higher chance of severe disability (aOR 1.40; 95% CI 1.38-41), morbidity (1.36; 1.33-1.38), and discharge to non-home (1.54; 1.52-1.56). OSA and GAD had higher odds of non-home discharge amongst post-AIS hospitalizations. Conclusion: DOCA is associated with poor outcomes among post-AIS patients. Prompt recognition by screening and timely management of DOCA may mitigate the adverse outcomes.

## Introduction

Stroke is the third leading cause of mortality in the world, and its prevalence only continues to increase [1]. According to the Centers for Disease Control and Prevention, each year, almost 800,000 Americans experience a single episode of stroke, with 76% of those being new episodes [2,3]. Those who experience a stroke will often be afflicted by chronic movement impairment as a result, leading to long-term disability [1,4]. Aside from physical disability, variables such as age, comorbidities, the severity of neurological deficit, and psychosocial factors (a support group or lack thereof) can all impact post-stroke quality of life (QOL) as well as hospital length of stay (LOS) [1,5].

There are many risk factors for stroke, such as increased blood pressure, type 2 diabetes, and cardiovascular diseases (CVD). Obstructive sleep apnea (OSA), as measured by the apnea-hypopnea index, is one such factor that significantly increases stroke risk by causing recurrent hypoxic states that activate the sympathetic nervous system during sleep, resulting in intrathoracic pressure changes and abnormal shifts in blood pressure [6-11]. These frequent physiological changes can cause damage associated with CVD, significantly increasing stroke risk [11]. Not only that, but severe cases of OSA can also impede post-stroke recovery by increasing the risk of neurological deficits [12].

Many individuals will also experience psychological side effects after a stroke episode, such as symptoms of depression, anxiety, and/or specific phobias, considerably decreasing their QOL [5,13-15]. Of those who experience a stroke, 31-35% will experience anxiety, and 28-37% will exhibit symptoms of depression within a two- to 10-year period post-stroke [16-19]. The pathophysiology behind post-stroke depression (PSD) is complex and remains poorly understood, but current literature suggests that serotonin reuptake inhibitors (SSRIs) may enhance motor functioning and neuroplasticity in recovering patients [20]. Similarly, little is known about the mechanisms behind post-stroke anxiety (PSA), although past studies have addressed the prevalence; one thing is certain: given the impact of PSD and PSA on stroke patients, there is a need to better understand the etiology in order to establish better treatment modalities.

Given how complicated the rehabilitation process following a stroke can be, it is important to examine how comorbid conditions such as depression, OSA, and concurrent anxiety (DOCA) may interact to impact a patient’s journey to recovery. In fact, OSA may be a modulator, increasing depressive symptoms and decreasing individuals’ cognitive and overall functioning [21,22]. To date, there is a severe lack of systematic reviews and/or meta-analyses identifying the QOL of post-stroke patients suffering from DOCA [20]. The aim of the study is to evaluate the prevalence and burden of DOCA in post-stroke hospitalization to create awareness of the importance of DOCA screening in order to ensure they receive effective treatment quickly. This effort will help with understanding how the interactions of comorbid conditions impact stroke recovery and how to address this issue.

## Materials and methods

We obtained the National Inpatient Sample (NIS) data from the Agency for Healthcare Research and Quality's Healthcare Cost and Utilization Project (HCUP) from January 2003 to December 2017. It is the largest publicly available all-payer inpatient care database in the United States, containing discharge-level data provided by HCUP states. This administrative dataset comprises information on roughly 8 million hospitalizations in 1,000 hospitals chosen to represent a 20% stratified sample of all community hospitals in the United States, covering more than 95% of the national population. NIS details can be found at http://www.hcup-us.ahrq.gov/db/nation/nis/nisdde.jsp. We adhered to the Strengthening the Reporting of Observational Studies in Epidemiology (STROBE) procedure and standards to carry out the study. The STROBE guidelines were developed to assist authors in effectively presenting their observational studies with high quality. It is important to note that these guidelines are not intended to validate the conducted study or serve as a framework for conducting an observational study. Rather, their purpose is to guide authors in presenting their research comprehensively and transparently.

Study population

We used the 9th and 10th revisions of the International Classification of Diseases, clinical modification code (ICD-9-CM and ICD-10-CM) to identify adult hospitalizations with a primary diagnosis of acute ischemic stroke (AIS) (ICD-9-CM code 577.8: ICD-10-CM code 577.8; sensitivity: specificity:). Among those AIS admissions, secondary diagnosis with major depressive depression (MDD) (ICD-9: 296.3 and 296.3 and ICD-10: F32.xx, F33.xx), OSA (ICD-9: 327.23 and ICD-10: G47.33), and GAD (ICD-9: 300.02 and ICD-10: F41.1) were identified using ICD-9 and 10-CM. We used ICD-9 and 10-CM codes to identify independent predictors (covariates), including the comorbidities of hypertension, diabetes mellitus, obesity, hypercholesterolemia, smoking, alcohol, drug abuse, epilepsy, tPA, and endovascular thrombectomy hemorrhagic transformation. Age <18 years and admissions with missing data for age, gender, and race were excluded. We have evaluated outcomes amongst patients with concurrent MDD, OSA, and GAD. The sample size was based on the available data.

Patient and hospital characteristics

The study focused on specific patient characteristics, including sex, age, race, insurance status, and concurrent diagnoses. Race was categorized as White (reference), African American, Hispanic, Asian or Pacific Islander, and Native American. Insurance status was classified as Medicare (reference), Medicaid, private insurance, and other/self-pay/no charge. The HCUP NIS database provides information on the total charges billed by hospitals for their services.

Outcomes

The primary objective of this study is to assess the prevalence and changes in prevalence trends of MDD, OSA, and GAD among patients with AIS from 2003 to 2017. A secondary goal was to examine the impact of concurrent MDD, OSA, or GAD on outcomes such as disability (loss of function), discharge destination, and hospital burden (length of hospital stay and cost) compared to patients without these conditions. The severity of disability/loss of function was evaluated using the All Patient Refined Diagnosis Related Group (APRDRG) severity scoring system. The software developed by 3M Health Information Systems was used to assign APRDRGs, with scores ranging from 1 (minor loss of function) to 4 (extreme loss of function). Discharge destination was categorized as either home or non-home (including short-term hospitals, skilled nursing facilities, or intermediate care facilities). Prolonged LOS and prolonged cost were defined as the LOS and cost exceeding the 90th percentile of the mean LOS and cost for AIS hospitalizations.

Statistical analysis

All statistical analyses were performed using the weighted survey methods in the statistical software suite (SAS) version 9.4 (SAS Institute, North Carolina, USA). The p-values of <0.05 were considered significant. Univariate analysis of differences between categorical variables was tested using the chi-square test, and analysis of differences between continuous variables (age, LOS, cost) was tested using unpaired student's t-test. Mixed-effects survey logistic regression models with weighted analysis were used to evaluate the outcomes of AIS with concurrent MDD, OSA, and GAD. We included demographics (age, gender, race), patient-level hospitalization variables (admission day, primary payer, admission type, median household income category), hospital-level variables (hospital region, teaching vs. nonteaching hospital, hospital bed size), comorbidities, and Elixhauser Comorbidity Index. The goodness of fit of the model was evaluated by the c-value.

## Results

Disease hospitalizations

We found a total of 6,577,146 AIS hospitalizations (unweighted 1,330,010) from 2003 to 2017, out of which 174,427 (2.65%), 203,868.8 (3.1%), and 287,204 (4.37%) had a secondary diagnosis of depression, OSA, and GAD, respectively. After excluding patients with <18 years and missing data for age, race, sex, and outcomes, the final cohort of AIS considered for the study was 5,690,773 with concurrent depression (163,226 (2.87%)), OSA (185,152 (3.25%)), and GAD (258,782 (4.55%)).

Demographics, patient and hospital characteristics, and comorbidities of patients with depression, OSA, and GAD

In AIS patients, females had a higher prevalence of depression (3.43% vs. 2.25% (males); p<0.0001) and GAD (5.92% vs. 3.04%) and a lower prevalence of OSA (2.21% vs. 4.40%) in comparison to males. Caucasians/Whites had a higher prevalence of depression (3.11% (Whites) vs. 2.12% (African Americans) vs. 2.84% (Hispanic) vs. 1.81% (Asians and Pacific Islanders) vs. 2.54% (Native American); p<0.0001), OSA (3.43% (Whites) vs. 3.27% (African Americans) vs. 2.42% (Hispanic) vs. 1.60% (Asian and Pacific Islanders) vs. 3.24% (Native American); p<0.0001], and GAD (5.26% (Whites) vs. 2.48% (African Americans) vs. 3.75% (Hispanic) vs. 2.13% (Asian and Pacific Islanders) vs. 3.59% (Native American); p<0.0001) in comparison to other races.

Medicare (67.36% vs. 59.48% vs. 65.66%; p<0.0001) and Medicaid (10.13% vs. 7.48% vs. 8.33%; p<0.0001) users had a higher prevalence of depression in comparison with OSA and GAD, respectively. The Midwest zone had a higher prevalence of all three DOCA components: depression (3.63% vs. 2.47% vs. 2.62% vs. 3.13%; p<0.0001), OSA (4.57% vs. 2.38% vs. 3.10% vs. 3.27%; p<0.0001), and GAD (5.33% vs. 4.19% vs. 4.66% vs. 3.92%; p<0.0001) in comparison to Northeast, South, and West, respectively. Diabetic patients had more prevalence of OSA (53% vs. 41% (depression) vs. 30% (GAD); p<0.0001), along with hypertensive patients (88% vs. 86% (depression) vs. 83% (GAD)), obese patients (14.64% vs. 4.78% (depression) vs. 5.28% (GAD)), patients with renal failure (25.26% vs. 23.83% (depression) vs. 14.97% (GAD)), ischemic heart disease (36.17% vs. 30.34% (depression) vs. 26.96% (GAD)), and atrial fibrillation (25.10% vs. 18.74% (depression) vs. 19.52% (GAD)) in comparison with other counterparts. Depression was more common in patients with hyperlipidemia (53.35% vs. 29.16% (OSA) vs. 22.96% (GAD)), and smoking (21.99% vs. 20.25% (depression) vs. 16.09% (OSA)) was more common in patients with GAD (Table 1).

**Table 1:** Characteristics of MDD, OSA, and GAD (DOCA) in patients with AIS MDD: major depressive depression, OSA: obstructive sleep apnea, GAD: generalized anxiety disorder, DOCA: depression, obstructive sleep apnea, and concurrent anxiety * Bed size of hospital indicates the number of hospital beds, which varies depending on hospital location (rural/urban), teaching status (teaching/non-teaching), and region (northeast/midwest/southern/western) The percentage in brackets indicates the direct comparison between MDD vs. OSA vs. GAD vs. neither of the three among AIS index hospitalizations

	MDD	OSA	GAD	Neither of DOCA	Total	p-value
Weighted (%)	163226 (3)	185152 (3)	258782 (5)	5083612 (89)	5690773 (100)	<0.0001
Demographics of patients						
Age mean (SD) (years)	69 (14)	66 (13)	69 (15)	71 (14)		<0.0001
Sex (%)						<0.0001
Male	61011 (48)	119427 (65)	82528 (32)	2451967 (48)	2714932 (48)	
Female	102175 (63)	65715.4 (36)	176234 (68)	2631328 (52)	2975452 (52)	
Race (%)						<0.0001
White	124486 (78)	137163 (75)	210079 (83)	3525910 (71)	3997638 (72)	
African American	19944 (13)	30803 (17)	23392 (9)	868311 (18)	942450 (17)	
Hispanic	12165 (8)	10349 (6)	16036 (6)	389242 (8)	427792 (8)	
Asian or Pacific Islander	2755 (2)	2432 (1)	3231 (1)	143472 (3)	151889 (3)	
Native American	670 (0.4)	853 (0.5)	945 (0.4)	23865 (0.5)	26333 (0.5)	
Characteristics of patients						
Median household income category for patient's zip code (%)						<0.0001
0-25th percentile	46960 (29)	51582 (28)	75743 (30)	1523394 (31)	1697679 (31)	
26-50th percentile	42565 (27)	47082 (26)	68879 (27)	1273959 (26)	1432485 (26)	
51-75th percentile	39379 (25)	45755 (25)	60733 (24)	1157363 (23)	1303229 (23)	
76-100th percentile	31916 (19)	37516 (21)	48799 (19)	1023830 (21)	1142060 (21)	
Primary payer (%)						<0.0001
Medicare	109843(67)	109935 (60)	169691 (66)	3394013 (67)	3783482 (67)	
Medicaid	16525 (10)	13833 (8)	21531 (8)	367450 (7)	419339 (7)	
Private insurance	28245 (17)	49724 (27)	50731 (20)	947614 (19)	1076314 (19)	
Other/self-pay/no charge	8448 (5)	11341 (6)	16468 (6)	366231 (7)	402488 (7)	
Admission type (%)						<0.0001
Non-elective	157084 (97)	178178 (97)	247706 (96)	4851379 (96)	5434347 (96)	
Elective	5745 (4)	6527 (4)	10442 (4)	220685 (4)	243399 (4)	
Admission day (%)						<0.0001
Weekday	122386 (75)	137917 (75)	194251 (75)	3781175 (74)	4235728 (74)	
Weekend	40840 (25)	47235 (26)	64531 (25)	1302438 (26)	1455045 (26)	
Characteristics of hospitals						
Bed size of hospital (%)*						<0.0001
Small	24513 (15)	22222 (12)	35947 (14)	649346 (13)	732028 (13)	
Medium	45685 (28)	49106 (27)	71821 (28)	1346727 (27)	1513339 (27)	
Large	92800 (57)	113149 (61)	150297 (58)	3071266 (61)	3427513 (60)	
Hospital location and teaching status (%)						<0.0001
Rural	12897 (8)	13198 (7)	29939 (12)	555276 (11)	611309 (11)	
Urban non-teaching	44564 (27)	58815 (32)	95830 (37)	1948463 (39)	2147671 (37)	
Urban teaching	105538 (65)	112464 (61)	132296 (51)	2563602 (51)	2913900 (51)	
Hospital region (%)						<0.0001
Northeast	28663 (18)	27657 (15)	48636 (19)	1056906 (21)	1161861 (20)	
Midwest	37559 (23)	47281 (26)	55144 (21)	893842 (18)	1033826 (18)	
South	63936 (39)	75710 (41)	113661 (44)	2185867 (43)	2439173 (43)	
West	33069 (20)	34505 (19)	41341 (16)	946999 (19)	1055913 (19)	
Comorbidities/confounders (%)						
Diabetes mellitus	66258 (41)	98821 (53)	76475 (30)	1766779 (35)	2008333 (36)	<0.0001
Hypertension	139649 (86)	163404 (88)	213584 (83)	4089538 (81)	4606174 (81)	<0.0001
Obesity	24736 (15)	75794 (41)	27312 (11)	389835 (8)	517681 (9.1)	<0.0001
Hyperlipidemia	87075 (53)	53982 (29)	59412 (23)	947991 (19)	1148460 (20)	<0.0001
Renal failure	38896 (24)	46772 (26)	38747 (15)	818890 (16)	943305 (17)	<0.0001
Smoking	33047 (20)	29782 (16)	56896 (22)	789672 (15)	909396 (16)	<0.0001
Alcohol	8436 (5)	6154 (3)	11382 (4)	202681 (4)	228653 (4)	<0.001
Drug abuse	8234 (5)	4374 (2)	9938 (4)	119338 (2)	141883 (3)	<0.001
Ischemic heart diseases	49529 (30)	69677 (36)	69778 (27)	1397086 (27)	1583370 (28)	<0.0001
Atrial fibrillation	30595 (19)	46478 (25)	50510 (20)	1148360 (23)	1275942 (22)	<0.0001

Prevalence of outcomes

Among AIS hospitalizations, patients with depression had a higher prevalence of morbidity (9% vs. 8% vs. 5% vs. 7%; p<0.0001), major or extreme loss of function (46% vs. 46% vs. 35% vs. 37%; p<0.0001), transfer to a skilled nursing facility (SNF)/intermediate care facility (ICF)/another type of facility (51% vs. 41% vs. 43% vs. 46%), home healthcare (16% vs. 14% vs. 15% vs. 14%), and lower prevalence of discharge to home (31% vs. 42% vs. 39% vs. 38%] in comparison with OSA, GAD, and non-DOCA patients, respectively (Table 2).

**Table 2:** Outcomes of MDD, OSA, and GAD among hospitalized patients with ischemic stroke APRDRG: All Patient Refined Diagnosis Related Group; SNF: skilled nursing facility; ICF: intermediate care facility; SE: standard error, MDD: major depressive depression, OSA: obstructive sleep apnea, GAD: generalized anxiety disorder, DOCA: depression, obstructive sleep apnea, and concurrent anxiety * Morbidity: LOS 10 days (> 90 percentile) AND discharge other than home The percentage in brackets indicates a direct comparison between FM vs. non-FM among migraineurs

	MDD	OSA	GAD	Neither of DOCA	Total
APRDRG severity or disability/loss of function (%)					<0.0001
Minor loss of function	8773 (6)	11827 (7)	30203 (12)	541134 (11)	
Moderate loss of function	77164 (49)	84800 (47)	133029 (53)	2460999 (51)	
Major loss of function	60786 (38)	67510 (38)	74514 (30)	1479340 (31)	
Extreme loss of function	12086 (8)	15239 (9)	12271 (5)	315301 (7)	
Total major/extreme loss of function (%)	72872 (46)	82749 (46)	86785 (35)	1794640 (37)	
Morbidity* (%)	14607 (9)	14043 (8)	13873 (5)	363667 (7)	< 0.0001
Discharge disposition (%)					< 0.0001
Routine/home	48487 (31)	74506 (42)	96681 (39)	1788942 (38)	
Transfer to a short-term hospital	4038 (3)	4982 (3)	6996 (3)	153209 (3)	
Transfer to SNF/ICF/another type of facility	80426 (51)	73826 (41)	106192 (43)	2173727 (46)	
Home healthcare	24444 (16)	25010 (14)	37319 (15)	655643 (14)	
Total discharge other than home (%)	108908 (69)	103818 (58)	150507 (61)	2982579 (63)	

Multivariable regression models for depression, OSA, and GAD among AIS hospitalized patients

We utilized a logistic regression model using the odds ratio (OR) to analyze the outcome prevalence of DOCA among AIS hospitalized individuals. The models assessed how DOCA influences the outcomes of disability in terms of APRDRG severity or loss of function, morbidity, and discharge to non-home facilities, respectively (Table 3).

**Table 3:** Multivariable regression analysis of outcomes of MDD, OSA, and GAD among hospitalized patients with ischemic stroke OR: odds ratio, CI: confidence interval, APRDRG: All Patient Refined Diagnosis Related Group, DOCA: depression, obstructive sleep apnea, and concurrent anxiety, MDD: major depressive depression, OSA: obstructive sleep apnea, GAD: generalized anxiety disorder * Disability: disability was defined by APRDRG loss of function and assigned using software developed by 3M Health Information Systems, where a score of 1 indicates minor loss of function, 2-moderate, 3-major, and 4-extreme loss of function $ Morbidity: morbidity was defined by the LOS > 10 days (90 percentile above the mean) AND discharge non-home # Discharge disposition was defined by discharge to home vs. non-home (short-term hospital, SNF, or ICF facility) Models were adjusted for demographics (age, gender, race), patient-level hospitalization variables (admission day, primary payer, admission type, median household income category), hospital-level variables (hospital region, teaching vs. nonteaching hospital, hospital bed size), and comorbidities (diabetes, hypertension, obesity, hyperlipidemia, renal dysfunction, smoking, alcohol, drug abuse, ischemic heart disease, atrial fibrillation)

	Model 1: APRDRG severity or loss of function*				Model 2: morbidity^$^				Model 3: Non-home discharge^#^			
	OR	95% CI		p-values	OR	95% CI		p values	OR	95% CI		p-values
No-DOCA	Reference											
MDD	1.40	1.38	1.41	< .0001>	1.36	1.33	1.38	< .0001>	1.54	1.52	1.56	< .0001>
OSA	1.21	1.20	1.22	< .0001>	0.97	0.95	0.99	0.0012	1.02	1.01	1.03	< .0001>
GAD	0.95	0.94	0.96	< .0001>	0.81	0.80	0.83	< .0001>	1.03	1.02	1.04	< .0001>
c-index	0.755				0.804				0.781			

Model 1: Regression Model Derivation for Disability (APRDRG Loss of Function)

The outcome of this model exhibited that depression increased the disability by 40% (OR= 1.40, 95% CI: 1.38-1.41, p<0.001) and OSA increased it by 21% (OR= 1.21, 95% CI: 1.20-1.22, p<0.001). Both depression and OSA displayed statistically significant effects. Though GAD also had statistical significance, it did not increase the outcome of disability (OR= 0.95, 95% CI: 0.94-0.96, p<0.001).

Model 2: Regression Model Derivation for Morbidity

Our second logistics regression model analyzed the prevalence of DOCA among hospitalized AIS patients OR. The presence of neither DOCA was used as the reference value. The results displayed that depression was the most prevalent among DOCA for AIS hospitalized patients (OR = 1.36, 95% CI: 1.33-1.38, p<0.001). While OSA (OR= 0.97, 95% CI: 0.95-0.99, p= 0.0012) and GAD (OR= 0.81, 95% CI: 0.80-0.83, p<0.001) were also statistically significant, neither of them displayed an increased chance of increasing among AIS patients.

Model 3: Regression Model for Non-home Discharge

This model revealed that all three variables of DOCA increased the outcome prevalence of patients being discharged to facilities other than their home (depression: OR= 1.54, 95% CI: 1.52-1.56, p<0.001; OSA: OR= 1.02, 95% CI: 1.01-1.03, p<0.001; and GAD: OR= 1.03, 95% CI: 1.02-1.04, p<0.001).

## Discussion

In our study, we examined the prevalence of depression, OSA, and anxiety in individuals with AIS. Our findings revealed that depression and OSA were associated with a higher probability of functional impairment in stroke patients, while the presence of anxiety also indicated an increased risk. The occurrence of additional health issues was more pronounced among AIS patients with depression. Moreover, we observed a higher prevalence of OSA in AIS patients with specific comorbidities, consistent with existing research linking CVD and OSA.

DOCA has been linked to a decline in QOL in the general population [23,24]. The stroke-specific QOL scale (SS-QOL) evaluates various aspects such as energy levels, engagement in family activities and social roles, speech or language impairment, mobility and functioning, mood and personality, self-care ability, and changes in vision, cognition, and productivity [23]. Difficulties or deteriorations in these areas can negatively impact the QOL of patients with AIS. Although depression, OSA, and anxiety are not explicitly included in these categories, they can still contribute to decreased energy levels, limited participation in activities, changes in mood and personality, and cognitive difficulties. Moreover, depression and OSA are known risk factors for coronary artery disease (CAD), and when depression coexists with CAD, the risk of mortality and morbidity increases [23]. Therefore, it is reasonable to expect that AIS patients with depression, OSA, or anxiety, in comparison to AIS patients without DOCA, will have a higher prevalence of morbidity, disability, and discharge to non-home facilities.

Previous studies have primarily focused on examining the prevalence of depression alone or compared it with OSA, cognitive impairment, and other factors in AIS patients [20]. The findings from these earlier studies consistently indicated that PSD negatively affected functional outcomes at three and six months [20]. Similarly, the presence of OSA or anxiety in AIS patients also had an impact on their functioning [25]. In our findings, we discovered that depression was linked to a 40% higher likelihood of impairing the loss of function in stroke patients, while OSA was associated with a 20% increased likelihood. However, the presence of anxiety did not demonstrate a significant increase in risk. It is worth noting that anxiety commonly coexists with depression [26], yet research on PSA is relatively limited compared to PSD [25]. Therefore, it is crucial to educate hospital staff about the importance of screening for both PSA and PSD, as both conditions have been shown to affect the functional outcomes of AIS patients.

Morbidity in AIS patients was increased only among the depressive population, having an increase of 36%. However, discharge to non-home was increased among all three variables of DOCA. This indicates that AIS patients with prevalent DOCA will most likely be discharged after their hospital stays to a non-home facility.

We sifted several noteworthy discoveries through our findings for sex, race, hospital location, hospital region, and comorbidities. Firstly, between males and females, depression was higher among females (68.1%), and OSA was higher among males (64.5%). Additionally, females had higher rates of depression (3.43%) and anxiety (5.93%), whereas OSA (4.4%) had the highest rate for males. Being male is one of the nonmodifiable risk factors in the development of OSA [27], but it could also be essential to see whether these males were obese or had other CVDs. Furthermore, our results showing a higher prevalence of depression among AIS patients is consistent with WHO findings. They reported the presence of gender bias during the treatment of mental health disorders. Doctors are more likely to diagnose a woman with depression, even if the men would display similar scores on the standardized measures [28]. In terms of seeking help, there is an apparent gender difference. Women were more likely to disclose their mental health issues with their primary care physician, whereas men were more likely to disclose them to a specialist [28]. Men are also more likely to report issues with alcohol than mood symptoms [28]. These findings prove that gender stereotypes continue to exist, even among physicians, which can contribute to men being self-conscious and avoiding seeking treatment. It is necessary to address and remove such barriers and stereotypes because they can hinder recovery in many patients, especially in patients with AIS.

Second, compared with other races, Caucasians had the highest rates for depression (3.11%), OSA (3.43%), and anxiety (5.26%), whereas Asians and Pacific Islanders had the lowest rate for depression (1.81%), OSA (1.60%), anxiety (2.13%). The cultural stigma around mental health issues is present among the Asian and Pacific Islander communities, which may result in underreporting of their mental health conditions [29]. Medical personnel assessing these symptoms must be aware of these stigmas and have the capability to approach these topics in these communities in a non-judgmental manner, especially as they are in a vulnerable state after suffering from an AIS.

Third, the Midwest region of the United States had the highest rates for DOCA (3.63%, 4.57%, and 5.33%, respectively). Similarly, Virani et al. [3] found that the Midwest region had the highest prevalence of cigarette smoking (16.9%), metabolic syndrome in adolescents (11.4%), the second-highest rate for obesity among adolescents (20.8%), one of the highest regions to have individuals with life-changing cardiovascular events. Another reported the prevalence of obesity, metabolic syndrome, and diabetes in the Midwest to be between 34.8%, 40%, and 9%, respectively, in the West North Central regions and 34.8%, 33.3%, and 8.9%, respectively, in the East North Central regions [30]. These reports are in alignment with our findings. For example, the self-medication hypothesis suggests that individuals cope with their depressive and anxiety symptoms by relying on smoking. Thus, depression and anxiety lead to smoking [31]. In turn, another hypothesis postulated that exposure to nicotine dysregulates the hypothalamic-pituitary axis. This can result in hypersecretion of cortisol and other neurotransmitters, which regulate individuals' responses to stress, leading to anxiety and depression [31]. Additionally, a significant risk factor for OSA is having a large neck circumference (NC) [32], which is closely associated with obesity. Thus, our findings of OSA having the highest prevalence in the Midwest region in AIS individuals can be attributed to the high prevalence of obesity. New research suggests that NC is a valuable predictor for general obesity (BMI) and particularly central obesity, as NC exposes the upper body subcutaneous fat [33]. Using this theory, physicians practicing in high OSA prevalent regions may want to use NC rather than BMI during OSA screening. In light of these findings, physicians in the Midwest should prioritize early screening, patient education, and awareness about the detrimental effects of smoking, mental health conditions, and OSA to improve the population's health outcomes.

Fourth, comorbid conditions such as diabetes (53%), hypertension (88%), renal failure (26%), ischemic heart disease (36%), and atrial fibrillation (25%) had a higher prevalence of OSA. In contrast, individuals with hyperlipidemia (53%), alcohol (5%), and drug abuse (5%) had a higher prevalence of depression, and smokers (22%) had a higher prevalence of anxiety. Hypertension, hyperlipidemia, diabetes, heart disease, and atrial fibrillation can fall under the category of CVD [27]. Our findings of a higher prevalence of OSA in AIS patients with these comorbidities follow the previously established associations between CVD and OSA. However, the fact that hyperlipidemia had a higher prevalence of depression than OSA should be an area of exploration for future researchers. Hyperlipidemia has been associated with elevated levels of systemic inflammatory markers, which have also been implicated in the development of depression among elderly patients [34]. Therefore, conducting a more detailed investigation into the relationship between depression and hyperlipidemia is crucial. Treating depression not only improves hyperlipidemia management but also mitigates the risk of AIS.

Finally, among DOCA, depression in AIS patients exhibited an increased risk across all three models, loss of function (40%), morbidity (36%), and non-home discharge (54%). A few randomized clinical trials have demonstrated that SSRIs, such as fluoxetine, have demonstrated a significant improvement in motor functioning [20]. Therefore, perhaps preventative treatment with SSRIs for AIS patients can potentially be the right way to move forward. This should be considered even when individuals suffering from AIS do not report depressive symptoms, as gender stereotypes and cultural stigma, as mentioned above, may deter people from reporting truthfully.

Our study is subject to several limitations. Firstly, it is important to acknowledge that the reliability and accuracy of our findings depend on the quality of data within the NIS database, which may have inherent limitations. Secondly, due to the cross-sectional design of the study, we cannot establish a causal relationship or examine the temporal sequence between DOCA and stroke outcomes. Additionally, the focus on hospitalizations in our study may introduce selection bias, potentially excluding stroke survivors who received care in outpatient settings or did not seek hospital treatment. This limitation could affect the generalizability of our prevalence estimates and their applicability to a broader population.

## Conclusions

Our study highlights the prevalence and impact of MDD, OSA, and GAD, collectively known as DOCA, on outcomes among individuals hospitalized for AIS. Study findings revealed that DOCA is common, with depression being the most prevalent, significantly contributing to poor discharge outcomes among stroke survivors. These results emphasize the importance of early recognition and management of DOCA through screening, as it can potentially mitigate adverse outcomes for post-AIS patients.
